# Ambient Air Pollution Exposure and Breast Cancer Risk Worldwide: A Systematic Review of Longitudinal Studies

**DOI:** 10.3390/ijerph21121713

**Published:** 2024-12-23

**Authors:** Jeeraporn Tippila, Naw Lah Say Wah, Kurnia Ardiansyah Akbar, Narumol Bhummaphan, Pokkate Wongsasuluk, Kraiwuth Kallawicha

**Affiliations:** 1 College of Public Health Sciences, Chulalongkorn University, Bangkok 10330, Thailand; 6574302953@student.chula.ac.th (J.T.); naw.candy@gmail.com (N.L.S.W.); ardiansyah_akbar@unej.ac.id (K.A.A.); narumol.b@chula.ac.th (N.B.); pokkate.w@chula.ac.th (P.W.); 2 College of Medicine and Public Health, Ubon Ratchathani University, Ubon Ratchathani 34190, Thailand; 3 Department of Occupational Health, Public Health Faculty, Jember University, Jember 68121, Indonesia

**Keywords:** outdoor air pollution, breast cancer, incident, epidemiology, carcinogenicity

## Abstract

Breast cancer is the most prevalent malignancy among women. Certain air pollutants have carcinogenic and estrogenic properties that can contribute to breast cancer development. This systematic review aimed to investigate the association between air pollution and breast cancer based on epidemiological evidence. This systematic review included articles published between 2013 and 2022 from Scopus and PubMed databases, focusing on cohort and nested case-control studies examining the association between outdoor air pollution and breast cancer. A total of 25 articles were included. A total of eight outdoor pollutants were analyzed, with seven showing a significant association with breast cancer risk. Specifically, the strong association between benzo[a]pyrene and breast cancer risk was reported. Furthermore, all four studies on nitrogen oxides (NO_x)_, fifteen out of eighteen (83.33%) on particulate matter less than 2.5 µm (PM_2.5_), nine out of thirteen studies (69.23%) on nitrogen dioxide (NO_2_), and three out of seven studies (42.86%) on particulate matter less than 10 µm PM_10_ showed an association with breast cancer risk (hazard ratio [HR]: 1.05–1.56; odds ratio [OR]: 1.03–1.86). In contrast, only one out of three studies (33.33%) on O_3_ (HR: 0.76–1.03) and all studies on cadmium (OR: 0.88–0.97) suggested a negative association with breast cancer risk. None of the studies on black carbon found an association with breast cancer risk. It is important to note the methodological limitations of this review, including potential publication bias due to the inclusion of only English-language articles and a regional focus on developed countries, which may limit the generalizability of findings. This study suggests that exposure to outdoor air pollutants is linked to an increased risk of breast cancer. Further research is needed to establish a causal relationship and the mechanisms by which environmental pollutants may trigger carcinogenic effects and contribute to breast cancer development through epigenetic pathways.

## 1. Introduction

Inevitably, humans inhale approximately 11,000 L of air daily, surpassing their food and drink intake [1]. The growing global alarm about air pollution has emerged as an urgent issue, with 99% of the world’s population inhaling air that exceeds the World Health Organization’s safety guidelines for harmful pollutants, especially particulate matter 2.5 (PM_2.5_) [2]. Despite the recommended international standard of 10 µg/m^3^, the recorded concentration in 2017 was alarmingly four times higher at 46 µg/m^3^, reflecting a pervasive and concerning trend [2].

Air pollution primarily originates from particulate matter (PM) and nitrogen oxides (NO_x_), including nitrogen dioxide (NO_2_). PM is categorized by aerodynamic diameter and includes PM_10_ and PM_2.5_ as common categories. Research indicates that exposure to PM_2.5_ is associated with adverse health outcomes, such as respiratory diseases, cardiovascular disorders, and increased mortality rates [3]. PM_2.5_ was classified as a Group 1 carcinogen by the International Agency for Research on Cancer (IARC), highlighting its potential contribution to lung cancer and other health issues. Historically, air pollution has been strongly linked to respiratory cancers, such as lung cancer, with a robust body of research demonstrating its role in increasing risk. The recognition of air pollution’s potential effects on non-respiratory cancers, such as colorectal cancer, oral cancer, and breast cancer, is a relatively recent development, underlining the novelty of this research focus [4,5,6].

Fossil fuel combustion, particularly in stationary power generation and combustion engine vehicles, is a major source of NO_x_ and NO_2_ [7]. While the direct carcinogenicity of NO_2_ is not definitely proven, it is a key indicator of diesel exhaust exposure, which is known to contain carcinogens like benzene, polycyclic aromatic hydrocarbons (PAHs), and PM. Traffic-related air pollution (TRAP), which includes PM and various pollutants, relies on NO_x_ and NO_2_ as indicators rather than their direct carcinogenic properties [8,9].

Annually, approximately 6.7 million premature deaths are attributed to the combined effects of ambient and household air pollution. According to the World Health Organization (WHO) report, outdoor air pollution alone was estimated to cause 4.2 million premature deaths globally in 2019, making it the second-highest risk factor for noncommunicable diseases [2]. These concerns are worsened by urbanization and industrialization, impacting social and economic development and highlighting the significant impact of air pollution on health and well-being.

Globally, breast cancer is one of the leading causes of mortality and morbidity among women [4]. Age, menstrual history, reproductive history, breastfeeding history, personal history such as physical activity, alcohol consumption, and hormonal use are the key recognized risk factors for breast cancer in women, apart from genetic factors and family history of the disease. The inheritability of breast cancer is thought to be between 5% and 10%, which suggests nongenetic factors are a part of the causes [9]. Various epidemiological studies have investigated the link between air pollution and breast cancer risk, a malignancy influenced by age, reproductive history, genetic factors, and environmental elements [10,11,12,13].

Environmental chemical factors, including bisphenol A, xenoestrogens, dioxins, and air pollution, play a critical role in breast cancer development [11,12]. Airborne pollutants exhibited estrogenic properties that may manipulate the estrogenic pathway in breast tissue regulation [10,11,13,14,15]. Recent studies have investigated the relationship between menopausal status, pollutants like PM or NO_2_, and the risk of breast cancer, emphasizing the complex interplay between hormonal factors and environmental exposures. Recent research is exploring the link between air pollution and specific subtypes of breast cancer, such as hormone-responsive positive and negative tumors, which make up approximately 70% of cases with positive estrogen or progesterone receptors [16,17,18,19].

While there is extensive research linking air pollution to lung cancer and other respiratory conditions, a gap remains in understanding its role in breast cancer, particularly in relation to specific pollutants and breast cancer subtypes. Addressing this gap is critical, given the estrogenic and carcinogenic properties of airborne pollutants, which may interact with breast tissue through hormonal and genetic pathways.

It is important to understand this relationship to elucidate the complex interplay between environmental exposures and the heterogeneity of breast cancer [20]. Globally, cancer resulted in nearly 10 million deaths in 2020, underscoring the need for a detailed examination of specific malignancies, such as breast cancer. As the most common malignancy worldwide, breast cancer accounted for 2.26 million cases and ranked as the fifth leading cause of cancer-related deaths, with 685,000 fatalities in 2020. Its prevalence, constituting 11.7% of new cancer cases and contributing to 6.9% of cancer-related deaths, highlights the urgency of understanding various risk factors, including potential environmental influences like air pollution [21].

This systematic review intends to explore the research gap on the linkage between air pollutants and breast cancer by thoroughly assessing longitudinal studies published between 2013 and 2022. Exploring the complex interplay between various air pollutants and breast cancer is crucial for advancing our knowledge of this critical health issue. By systematically reviewing and analyzing existing literature, this study sought to substantially contribute to the up-to-date body of evidence on specific pollutants and their potential impact on the incidence and subtypes of breast cancer. The valuable insights gained from this investigation offer guidance for future research, public health initiatives, and directed interventions that reduce the incidence of breast cancer due to outdoor air pollution exposure.

## 2. Materials and Methods

### 2.1. Search Strategy and Inclusion Criteria

The review was conducted following the Preferred Reporting Items for Systematic Reviews and Meta-Analyses (PRISMA) guidelines. The association between air pollution and breast cancer was explored using comprehensive data from Scopus and PubMed databases. The study was focused on the primary keywords “air pollution” and “breast cancer”, with an emphasis on “outdoor air pollution” and its effects on breast cancer. Articles published in English between 2013 and 2023 focusing on female subjects, employing cohort and nested case-control study designs, and investigating the linkage between outdoor air pollution and breast cancer were included; systematic reviews, qualitative studies, ecological studies, and studies of industrial emissions were excluded.

**Figure 1:**
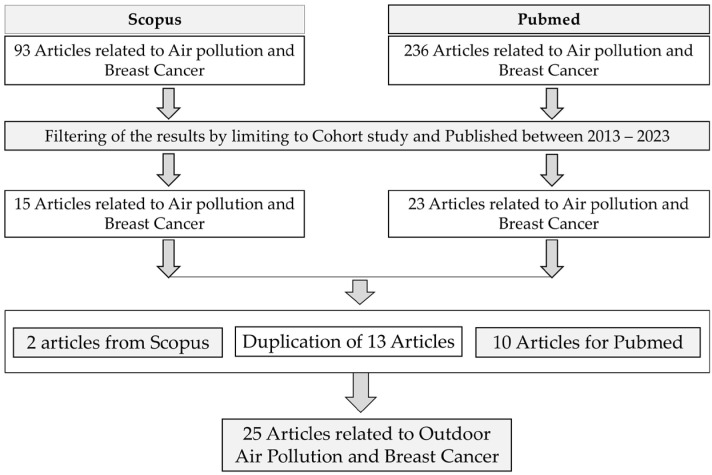
The article selection process following PRISMA guidelines.

The term “outdoor air pollutant” in this study refers to atmospheric air containing one or more substances at concentrations and durations that exceed natural limits [22]. A thorough search for relevant articles was conducted using the Scopus and PubMed databases, with keywords such as “Outdoor Air Pollution”, “PM_2.5_”, “PM_10_”, “NO_2_”, “NO_x_”, “O_3_”, “Cadmium”, “Black Carbon”, “Benzo[a]pyrene (BaP)”, “Breast Cancer”, and “Incidence”. Initially, 329 articles were identified, 93 from Scopus and 236 from PubMed. After applying inclusion and exclusion criteria, 38 articles were shortlisted for detailed analysis. There were 13 duplicate articles identified, resulting in the final inclusion of 25 unique articles from the two primary databases. The entire selection process is depicted in Figure 1, ensuring transparency and methodological clarity in the article retrieval and screening process.

### 2.2. Data Analysis

All 25 articles included in the systematic review met the inclusion criteria and underwent appraisal using the National Heart, Lung, and Blood Institute (NHLBI) assessment tool for observational cohort and cross-sectional studies. The quality of a systematic review hinges on the authors’ ability to interpret findings accurately and describe methods clearly [23]. To enhance methodological rigor, every author assessed the quality of each study. Discrepancies in the quality scores were discussed and resolved through consensus, with input from another reviewer when necessary. The NHLBI appraisal tool was selected for its suitability to various study designs and rigorous evaluation of methodological errors. The tool assigns a score ranging from 0 to 14, and only articles with a score above 10 were included in the study [24].

## 3. Results

### 3.1. Study Characteristics

The review included 25 articles that passed the quality assessment criteria (NHLBI score above 10). The majority of the research was conducted in North America, followed by Europe and Asia, with adults and older adults being the primary focus. Generally, the articles included in this systematic review were longitudinal studies with follow-up periods ranging from 3 to 22 years. The largest study explored the linkage between ambient air pollution and the risk of breast cancer in Canada, utilizing data from the Ontario Cancer Registry. This cohort included 4,952,022 individuals enrolled in the insurance plan since 1996. The distribution of general characteristics of the studies included in this review is presented in Table 1.

### 3.2. Pollutants Analyzed

A total of eight pollutants were analyzed: PM_2.5_, NO_2_, PM_10_, NO_x_, O_3_, cadmium, BaP, and black carbon. The most frequently studied pollutants were PM_2.5_ (72.00%), NO_2_ (52.00%), and PM_10_ (28.00%). Among these, specific subtopics were addressed, such as mammographic breast density (three articles), breast cancer risk among premenopausal and postmenopausal women in Spain (one article), and traffic-related air pollution (TRAP) (two articles). The detailed information for these articles is presented in Table 2.

### 3.3. Findings by Pollutant

Particulate Matter (PM_2.5_ and PM_10_): PM_2.5_ was the most studied pollutant, with 15 out of 18 studies (83.33%) reporting a significant association with breast cancer risk (hazard ratio [HR]: 1.05–1.56). Similarly, three out of seven studies (42.86%) on PM_10_ indicated an association (HR: 1.03–1.29).

Nitrogen Dioxide (NO_2_) and Nitrogen Oxides (NO_x_): Nine out of thirteen studies (69.23%) on NO_2_ and all four studies on NO_x_ reported a significant association with breast cancer risk (HR: 0.76–1.86 for NO_x_, 1.03–1.44 for NO_2_).

BaP and Black Carbon: BaP showed the strongest association with breast cancer risk. In contrast, none of the studies on black carbon identified a significant association.

Ozone (O_3_) and Cadmium: Only one out of three studies (33.33%) on O_3_ suggested a negative association with breast cancer risk (HR: 0.76–1.03), as did all studies on cadmium (odds ratio [OR]: 0.88–0.97).

### 3.4. General Trends

Out of the 25 articles reviewed, 24 reported a link between outdoor air pollutants and breast cancer risk, while one study found no such association. Most of the research focused on older adults. Seven of the eight analyzed pollutants demonstrated a significant association with breast cancer risk, highlighting BaP, PM_2.5_, NO_x_, and NO_2_ as the most strongly linked to increased risk.

## 4. Discussion

This review of 25 studies, spanning from 2013 to 2023, offers important insights into the link between outdoor air pollution and breast cancer risk. A key finding is the varied influence of different pollutants on breast cancer risk. Benzo[a]pyrene (BaP), a polycyclic aromatic hydrocarbon (PAH) commonly found in outdoor air pollution, particularly from the combustion of fossil fuels and organic materials, is classified as a Group 1 carcinogen by IARC, indicating its carcinogenic potential in humans. The link between BaP and breast cancer is believed to arise from its competence to cause DNA damage and generate reactive oxygen species, leading to oxidative stress and genomic instability—both key factors in cancer development. BaP’s primary mode of action is known to be direct DNA damage caused by the production of DNA adducts, which results in DNA mutations and lesions.

Furthermore, BaP can interfere with normal cellular functions, such as cell cycle regulation and apoptosis, promoting uncontrolled cell growth and tumor development. Research indicates that prolonged exposure to BaP may elevate the risk of breast cancer, emphasizing the need to reduce exposure to this harmful pollutant for public health protection [28,29,31].

The majority of studies focusing on PM_2.5_ and PM_10_ have shown a significant association with breast cancer. Notably, a previous study emphasized the potential role of PM_2.5_ and PM_10_ exposure in breast cancer [46]. Nevertheless, it is important to note that not all studies have revealed a statistically significant effect of PM exposure. These differing results highlight the complexity of the relationship between PM and breast cancer, indicating the need for further investigation into potential contributing factors [47].

Studies investigating NO_2_ and NO_x_ exposure in traffic-concentrated areas, such as Amadou et al. (2023), consistently reported an association with breast cancer [36]. The reason for this link lies in the composition of TRAP. NO_2_, a major component of TRAP, contributes to the formation of harmful pollutants like PM. These pollutants are known to induce oxidative stress, inflammation, and genotoxicity, all of which are known precursors to cancer development. Additionally, proximity to traffic-emitting sources enhances an individual’s exposure to a complex mixture of carcinogens, including PAHs and volatile organic compounds, further raising the risk of adverse health outcomes, including breast cancer [48].

The significance of the association between outdoor air pollutants and breast cancer risk is underscored by studies that stratify based on hormone receptor status, particularly estrogen receptor-negative (ER−) tumors, and menopausal status. Several studies in this review found a higher risk of breast cancer linked to exposure to pollutants such as PM_2.5_, NO_2_, and NO_x_ among women with ER− tumors. This is crucial as ER− tumors are often more aggressive and have poorer outcomes compared to ER-positive tumors. Furthermore, the review reported an increased risk of breast cancer associated with pollutant exposure among postmenopausal women. This is important because menopausal status can influence hormone levels and receptor activity, potentially modifying the influence of environmental pollution exposure on the development of breast cancer. Stratifying results by hormone receptor status and menopausal status provides a more in-depth understanding of the connection between air pollution and breast cancer. Therefore, targeted interventions and further research in specific subgroup populations are needed to mitigate the impact of outdoor air pollutant exposure on breast cancer risk [49].

The unexpected finding of a potential negative association between O_3_ and cadmium and breast cancer in some studies requires further investigation. Ozone, a major component of smog and a harmful air pollutant known for its detrimental effects on respiratory health showed a surprising potential protective effect against breast cancer in a few studies. This may be attributed to its oxidative properties potentially inducing cell apoptosis in certain conditions, although this hypothesis warrants further validation. Moreover, variations in exposure levels, study populations, or potential protective co-exposures to other pollutants might have influenced these findings. This contrasts with the well-known adverse health effects of O_3_ exposure. Similarly, cadmium, a toxic heavy metal present in industrial emissions, tobacco smoke, and certain foods, is typically linked to various health risks, including cancer. However, the review found no significant association between cadmium exposure and breast cancer risk. One possible explanation could be variations in cadmium bioavailability due to dietary differences, genetic susceptibility, or other environmental exposures. Additionally, misclassification of cadmium exposure or underreporting in some studies might contribute to this finding. These findings raise questions about the underlying mechanisms and potential confounding factors influencing the relationship among O_3_, cadmium, and breast cancer. Addressing these complexities requires future studies that incorporate detailed exposure assessments, consider gene–environment interactions, and control for potential confounders. Further research is necessary to unravel these complex interactions and determine the true nature of the association among O_3_, cadmium, and breast cancer risk [50].

The studies included in this review employed various analysis techniques, such as multivariable regression models, Cox proportional hazards models, and mixed-effects models, to estimate the association between air pollution and breast cancer. These methods each have strengths and limitations. For example, Cox models are widely used for longitudinal data to handle time-to-event outcomes but rely on assumptions about proportional hazards, which may not always hold. Mixed-effects models account for clustered or hierarchical data structures, offering flexibility but increasing computational complexity. While these methods enhance the robustness of findings, differences in statistical techniques, adjustment variables, and exposure assessment methods across studies could partially explain the variability in results. It is crucial for future studies to standardize methodologies where feasible, improving comparability and reliability across research efforts.

Notably, our review revealed that all studies on black carbon found no significant association with breast cancer risk, which is a noteworthy finding. Black carbon, a component of fine PM (PM_2.5_) primarily emitted from combustion processes, is known for its hazardous effects, particularly on respiratory health. However, the lack of a significant association with breast cancer risk suggests a nuanced relationship between black carbon exposure and breast cancer development. It is plausible that the mechanisms through which black carbon affects respiratory diseases differ from those involved in breast cancer. Additionally, the limited number of studies directly investigating the association between black carbon and breast cancer may contribute to the uncertainty in the findings [8,40].

While existing research has extensively linked air pollution, particularly PM_2.5_ and NO_2_, to lung cancer, the association with breast cancer has received less attention. Our review highlights the need to better understand the mechanisms underlying the impact of air pollution on breast cancer risk. Future research should explore potential pathways, considering the complex interplay of hormonal factors, genetic predispositions, and environmental exposures. Given the limited evidence on black carbon, further research is required to ascertain its role in breast cancer development [50].

Although this review covered a wide range of pollutants, the review did not find other common pollutants, such as sulfur dioxide and carbon monoxide, that were associated with breast cancer. Further studies on other common pollutants could be considered. Moreover, while this review exclusively examines outdoor air pollution, the potential interaction between indoor and outdoor air pollutants remains underexplored. Indoor air pollutants, such as volatile organic compounds (VOCs), cooking smoke, and other household pollutants, may exacerbate the effects of outdoor pollutants or act as independent risk factors for breast cancer [51,52]. The exclusion of indoor air pollution from this review limits its scope but opens avenues for future research to address the joint influence of both indoor and outdoor pollutants on breast cancer risk.

This systematic review, based on longitudinal studies with robust follow-up periods and considerable study populations, provides a comprehensive overview of the current literature. Including studies from 2013 to 2023 ensures the collection of up-to-date information on this evolving topic. It is important to recognize that this study offers novelty by focusing exclusively on longitudinal designs, which are crucial for establishing temporal relationships and reducing biases compared to cross-sectional studies. Additionally, the emphasis on specific outdoor air pollutants, including PM_2.5_, NO_2_, PM_10_, Nox, O_3_, Black carbon, cadmium, and benzo[a]pyrene (BaP), and their role in breast cancer risk fills a critical gap in existing literature, as prior reviews often combine outdoor and indoor pollutants without disaggregating their effects [15] and some previous systematic review articles focused on NO_2_, PM_2.5_, and PM_10_ [53], which is less than the coverage of the current review. Nevertheless, there are limitations to this review. The exclusion of unpublished studies or those in non-English languages may lead to publication bias, which could impact the generalizability of the results. Furthermore, the focus on developed countries in America and Europe limits the global scope of the review. The lack of data from developing regions, where air pollution exposure patterns may differ significantly, is a critical area for future exploration. To improve our understanding of the link between air pollution and breast cancer, future research should incorporate more diverse datasets from a broader range of regions, as well as broader search terminology. Additionally, this review focused on the primary organic aerosols that are directly released from sources and not on the studies related to secondary organic aerosols (SOAs), which are formed through the oxidation of pollutants in the atmosphere [54]. SOAs could be reviewed in the future.

## 5. Conclusions

In conclusion, our systematic review highlights a discernible association between outdoor air pollutants and the risk of breast cancer, emphasizing the need for further investigation to establish causality. Specifically, pollutants such as BaP, PM_2.5_, PM_10_, NO_2_, NO_x_, O_3_, and cadmium exhibit significant associations, particularly in analyses stratified by hormone receptor (ER−) and menopausal status, indicating varying effects across different subgroups. This suggests a complex interaction between environmental pollutants and breast cancer development. These findings have significant implications for public health policy, underscoring the urgent need for stricter air quality regulations, enhanced monitoring of pollution levels, and community-driven initiatives to reduce exposure, particularly in urban and industrialized regions. Public health strategies should prioritize awareness campaigns on the carcinogenic risks of air pollution while promoting preventive measures to mitigate these risks. Future research should adopt standardized exposure assessment methods, conduct longitudinal studies with diverse populations, and utilize advanced molecular techniques, such as epigenomic and transcriptomic analyses, to unravel the biological mechanisms and gene–environment interactions underlying the association between air pollutants and breast cancer. A multidisciplinary approach integrating epidemiological, molecular, and environmental perspectives is essential for advancing our understanding of these relationships and informing effective public health strategies to reduce breast cancer incidence.

## Figures and Tables

**Table 1:** Characteristics of included studies evaluating the association between outdoor air exposure and breast cancer.

Variables	Number	Percent
Region		
Asia	4	16.00
Europe	9	36.00
North America	11	44.00
South America	1	4.00
Age range		
Youth (17–24 years)	1	3.85
Adults (25–64 years)	6	23.08
Older adults (45–64 years)	9	34.62
Elderly (over 65 years)	1	3.85
Older adults and the elderly (>40 years)	5	19.23
Not applicable	4	15.38
Pollutants		
PM_2.5_	18	72.00
NO_2_	13	52.00
PM_10_	7	28.00
NO_x_	4	16.00
O_3_	3	12.00
Black carbon	2	8.00
Cadmium	1	4.00
Benzo[a]pyrene (BaP)	1	4.00

*N* = 25 articles.

**Table 2:** Summary of studies on breast cancer risk associated with outdoor air pollution.

Article Code, Author	Country	Population	Age Group (Years)	Pollutants	Follow-Up Period (Years)	Statistical Analysis	Estimate Risk (Hazard Ratio [HR] and Odds Ratio [OR])
A1, White et al. (2021) [25]	USA	59,000	21–69	PM_2.5_, NO_2_, and O_3_	Mean = 18.3 years	Cox proportional hazards regression	PM_2.5_ Overall breast cancer HR of 1.18; 95% CI: 1.00–1.39, stratified by geographic residency in the Midwest HR of 1.53; 95% CI: 1.07–2.19, stratified by geographic residency in the Midwest and ER− HR of 1.32; 95% CI: 1.03–1.71, stratified by geographic residency in the Midwest and premenopausal status
A2, Cheng et al. (2020) [16]	USA	57,589	45–75	NO_x_, NO_2_, PM_10_, and PM_2.5_	14.7 ± 4.3 years	Cox proportional hazards models	HR of NO_x_ = 1.35; 95% CI: 1.02–1.79 HR of NO_2_ = 1.44; 95% CI: 1.04–1.99 HR of PM_10_ = 1.29; 95% CI: 1.07–1.55 HR of PM_2.5_ = 1.85; 95% CI: 1.15–2.99, stratified by race/ethnicity and determined using kriging HR of NO_x_ = 1.21 (95% CI: 1.01–1.45) HR of NO_2_ = 1.26; 95% CI: 1.00–1.59, stratified by race/ethnicity and determined using land use regression
A3, Niehoff et al. (2022) [26]	USA	48,453	N/A	NO_2_ and PM_2.5_	Median = 10.9 years	Cox proportional hazards models	HR of NO_2_ = 1.28; 95% CI: 1.05–1.56, stratified by BOADICEA score above the 90th percentile HR of NO_2_ = 1.12; 95% CI: 1.00–1.25, stratified by BOADICEA score above the median and ER+
A4, Terre-Torras et al. (2022) [27]	Spain	1,798,838	17–85	PM_2.5_, PM_10_, and NO_2_	Median = 10 years	cause-specific Cox Proportional Hazards models	HR of PM_2.5_ = 1.03; 95% CI: 1.01–1.04 HR of PM_10_ = 1.03; 95% CI: 1.01–1.05 HR of NO_2_ = 1.05; 95% CI: 1.02–1.08, stratified by postmenopausal status
A5, White et al. (2019) [28]	USA	47,433	35–74	PM_2.5_, PM_10_, and NO_2_	Mean = 8.4 years	Cox proportional hazards regression	In overall breast cancer: HR of PM_2.5_ = 1:05; 95% CI: 0.99–1.11 HR of NO_2_ = 1.06; 95% CI: 1.02–1.11 Invasive breast cancer stratified by geographic region: HR of PM_2.5_ = 1:14; 95% CI: 1.02–1.27, in the western United States HR of NO_2_ = 1:16; 95% CI: 1.01–1.33, in the southern United States
A6, Amadou et al. (2020) [29]	France	8118	40–65	Cadmium	5–9 years	Conditional logistic regression models	OR Q_5_ vs. Q_1_ = 0.63; 95% CI: 0.41–0.9, stratified by ER− OR Q_4_ vs. Q_1_ = 0.62; 95% CI: 0.40–0.95, stratified by ER− and progesterone receptor-negative (PR−) breast tumors
A7, Yaghjyan et al. (2017) [8]	USA	279,967	≥40	PM and O_3_	9 years	Polytomous logistic regression	Among women with heterogeneously dense vs. scattered fibro glandular breasts OR of PM_2.5_ (fourth vs. first quartile) = 1.19 95% CI: 1.16–1.23 OR of O_3_ = 0.80; 95% CI: 0.73–0.87
A8, Ou et al. (2020) [30]	USA	15,903	<39	PM_2.5_	5 and 10 years postdiagnosis	Multivariable Cox models	HR of 5-year = 1.50; 95% CI: 1.29–1.74 HR of 10-year = 1.30; 95% CI: 1.13–1.50, stratified by PM_2.5_ levels and follow-up periods post-diagnosis
A9, Bai et al. (2020) [31]	Canada	4,952,022	35–85	PM_2.5_, NO_2_, O_3_, and O_x_	15 years	Cox proportional-hazards models	In a sensitivity analysis involving a 10-year lag in exposure, where rigorous observation was employed: HR of PM_2.5_ = 1.02; 95% CI: 1.00–1.03 HR of NO_2_ = 1.03; 95% CI: 1.01–1.05
A10, Amadou et al. (2021) [32]	France	10,444	40–65	Benzo[a]pyrene (BaP)	22 years	Multivariable conditional logistic regression models	OR per 1 IQR = 1.20; 95% CI: 1.03–1.40, stratified by premenopausal status ORs = 1.17; 95% CI: 1.04–1.32, stratified by hormone receptor status (ER+) OR = 1.16; 95% CI: 1.01–1.33, stratified by PR+ OR = 1.17; 95% CI: 1.01–1.36, stratified by ER+ PR+ breast cancer
A11, Cohen et al. (2019) [33]	Israel	10,627	Mean: 71.3	NO_x_	10 years	Cox regression models	HR of NO_x_ = 1.56; 95% CI: 1.13–2.15, with a NO_x_ cutoff of ≥ 25 ppb cutoff
A12, Reding et al. (2015) [34]	USA	47,591	35–74	NO_2_, PM_2.5_, and PM_10_	10 years	Cox proportional hazards and polytomous logistic regression	RR of NO_2_ = 1.10; 95% CI: 1.02–1.19, stratified by ER+
A13, Cohen et al. (2018) [35]	Israel	12,784	Mean: 68	NO_x_	10 years	Cox regression models	HR of NO_x_ =1.43; 95% CI: 1.12–1.83
A14, Amadou et al. (2023) [36]	French	8995	40–65	NO_2_	20 years	Multivariable conditional logistic regression models	Overall OR of NO_2_ = 1.09; 95% CI: 1.01–1.18 OR = 1.10; 95% CI: 1.01–1.21, stratified by postmenopausal status
A15, Hvidtfeldt et al. (2023) [37]	Europe	199,719	Mean: 49.0	NO_2_, PM_2.5_, and black carbon	20 years	Cox proportional hazards models	HR of NO_2_ = 1.03; 95% CI: 1.00–1.06 HR of PM_2.5_ = 1.06; 95% CI: 1.01–1.11
A16, Huynh et al. (2015) [7]	Denmark	4769	50–69	NO_x_ and NO_2_	8 years	Logistic regression	OR of NO_x_ = 0.96; 95% CI: 0.93–1.01, per 20 μg/m^3^ OR of NO_2_: = 0.89; 95% CI: 0.80–0.98, per 10 μg/m^3^ of NO
A17, Tagliabue et al. (2016) [38]	Italy	2021	51–69	PM_2.5_	3 years	Multivariable Cox proportional hazards model	HR of quartile II PM_2.5_ = 1.82; 95% CI: 1.15–2.89 HR of quartile III = 1.73; 95% CI: 1.12–2.67 HR of quartile IV = 1.72; 95% CI: 1.08–2.75
A18, Goldberg et al. (2019) [39]	Canada	89,835	40–59	NO_2_	3 years	Cox proportional hazards model	In premenopausal women, the rate ratio (RR) = 1.17; 95% CI: 1.00–1.38 for an age cutoff of 52 years
A19, Andersen et al. (2017) [40]	Denmark	22,877	>44	PM_2.5_, PM_10_, and NO_2_	13 years	Time-varying Cox proportional hazards regression to model	HR of PM_2.5_ = 0.99; 95% CI: 0.94–1.10, per interquartile range of 3.3 mg/m^3^ HR of PM_10_ = 1.02; 95% CI: 0.94–1.10, per 2.9 mg/m^3^ HR of NO_2_ = 0.99; 95% CI: 0.93–1.05, per 7.4 mg/m^3^
A20, Hart et al. (2016) [18]	USA	115,921	25–42	PM_10_ and PM_2.5_	10 years	Time-varying Cox proportional hazard model	HR of PM_2.5_ = 0.76; 95% CI: 0.61–0.95
A21, Kotake et al. (2023) [41]	Japan	44,280 mammography	Mean: 62	PM_2.5_	10 years	A logistic marginal model	OR in 1-year exposure of PM_2.5_ = 1.027; 95% CI: 1.019–1.034 OR in 3-year exposure = 1.029; 95% CI: 1.022–1.036 OR in 5-year exposure = 1.044; 95% CI: 1.037–1.052
A22, Hu et al. (2013) [42]	USA	255,128	>25	PM_10_ and PM_2.5_	10 years	Marginal cox proportional hazards models	HR of PM_10_ = 1.13; 95% CI: 1.02–1.25 HR of PM_2.5_ = 1.86; 95% CI: 1.12–3.10
A23, Prada et al. (2021) [43]	Mexico	151	>18	PM_2.5_	8 years	Linear and logistic regression models	RR of PM_2.5_ = 1.32; 95% CI: 1.04–1.674
A24, Poulsen et al. (2023) [44]	Denmark	55,745	<55	PM_2.5_, back carbon, and NO_2_	14 years	Conditional logistic regression	OR of PM_2.5_ =1.21; 95% CI: 1.11–1.33
A25, Huang et al. (2022) [45]	Taiwan	407,415	>20	PM_2.5_	15 years	Cox proportional hazards models	HR of PM_2.5_ = 1.12; 95% CI: 1.03–1.22.

## Data Availability

All data used in this study are presented within the article.
